# Association of physiological reserve measures with adverse outcomes following liver transplantation

**DOI:** 10.1002/jgh3.12702

**Published:** 2022-01-12

**Authors:** James S Kimber, Richard J Woodman, Sumudu K Narayana, Libby John, Jeyamani Ramachandran, David Schembri, John W C Chen, Kate R Muller, Alan J Wigg

**Affiliations:** ^1^ Faculty of Health and Medical Sciences University of Adelaide Adelaide South Australia Australia; ^2^ College of Medicine and Public Health Flinders University of South Australia Adelaide South Australia Australia; ^3^ Hepatology and Liver Transplant Unit Flinders Medical Centre Adelaide South Australia Australia; ^4^ South Australian Liver Transplant Unit Flinders Medical Centre Adelaide South Australia Australia; ^5^ Respiratory Function Unit Flinders Medical Centre Adelaide South Australia Australia

**Keywords:** cardiopulmonary exercise testing, handgrip strength, hospital length of stay, intensive care length of stay, sepsis

## Abstract

**Background and Aim:**

The comparative utility of physiological reserve measures in predicting important clinical outcomes following liver transplantation (LT) requires further study. The aim of this work was therefore to compare the utility of physiological reserve measures in predicting early adverse clinical outcomes post‐LT.

**Methods:**

A single‐center, retrospective cohort study of LT patients consecutively recruited between 1 January 2015, and 31 August 2020. Outcomes measured were sepsis and death within 12 months of LT, hospital length of stay (LOS), and intensive care LOS. Physiological reserve measures were handgrip strength, mid‐arm muscle circumference, and cardiopulmonary exercise testing (CPET) measures. Analysis was performed using univariate and multivariate logistic regression for sepsis and death, and univariate and multivariate Cox regression for hospital and intensive care LOS.

**Results:**

Data were obtained for 109 subjects. Patients were predominantly (64%) male with a median (interquartile range [IQR]) age of 57 (49–63) and median (IQR) Model for End‐Stage Liver Disease score of 16 (11–21). In multivariate analysis, the odds of sepsis were lower in patients in the highest *versus* lowest tertile (odds ratio = 0.004; 95% confidence interval [CI] 0.00–0.13; *P* = 0.002). Hospital LOS was linearly associated with handgrip strength (hazard ratio [HR] = 1.03; 95% CI 1.00–1.06; *P* = 0.03) in multivariate analysis. Intensive care LOS was associated with peak VO_2_ (HR 1.83; 95% CI 1.06–3.16; *P* = 0.03) and V_E_/VCO_2_ slope (HR 0.71; 95% CI 0.58–0.88; *P* = 0.002) in multivariate analysis.

**Conclusion:**

Handgrip strength and CPET both identify candidates at high risk of adverse outcomes after LT.

## Graphical abstract

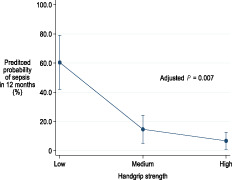

The authors aimed to compare the associations between the physiological reserve measures handgrip strength, mid‐arm muscle circumference, and cardiopulmonary exercise testing (CPET) with early adverse clinical outcomes post LT. A single‐centre, retrospective cohort was performed with LT patients recruited consecutively between 2015 and 2020. Handgrip strength and CPET both identify candidates at high risk of adverse outcomes post LT.

## Introduction

The prediction of important clinical outcomes (sepsis, prolonged inpatient and intensive care stay, mortality) following liver transplantation (LT) remains complex and challenging and is likely to be determined by multiple factors. Knowledge of the importance of physiological reserve and its impacts on clinical outcomes has been generated initially from the geriatric and surgical literature.

Frailty is defined as “a state of vulnerability to poor resolution of homoeostasis after a stressor event and is a consequence of cumulative decline in many physiological systems during a lifetime”.^1^ It has been shown to be associated with outcomes in a number of chronic diseases, including chronic liver disease.^2^, ^3^, ^4^ Frailty can be assessed in a number of ways including subjective measures (Karnofsky performance status, Eastern Cooperative Oncology Group, activities of daily living, Fried Frailty Index) and more complex objective measures (6‐min walk test, gait speed, the Liver Frailty Index, Short Physical Performance Battery) and all of these measures have demonstrated associations with adverse pre‐ and/or post‐LT outcomes.^5^ Handgrip strength, which is one component of the Liver Frailty Index, is a very simple bedside test that by itself has demonstrated an association with survival in chronic liver failure^6^, ^7^ and with LT wait‐list mortality.^8^

Sarcopenia, a term that describes the loss of muscle mass and strength with increasing age,^9^ is a related concept to frailty.^10^, ^11^, ^12^, ^13^ It describes objective measures of muscle mass and quality, rather than functional outcomes. Measures of sarcopenia are objective and reproducible.^3^, ^14^, ^15^ Rather than reflecting acute severity of illness, these measures of muscle mass are likely chronic indicators of overall health.^3^ Sarcopenia measures have also been shown to predict wait‐list mortality, independently of liver disease severity.^16^ In addition, sarcopenia has been associated with a number of other important clinical outcomes in LT candidates, post‐LT mortality, prolonged hospital and intensive care unit (ICU) stay, and risk of serious infection post‐transplantation.^17^, ^18^, ^19^, ^20^ One drawback of these measures is requirement for cross‐sectional imaging and software to calculate muscle mass, which may not be widely available. Anthropometric measurements, such as mid‐arm muscle circumference (MAMC), are highly related to lean muscle mass^20^ and have also been shown to be useful in predicting mortality.^21^ MAMC may therefore provide a simpler form of sarcopenia assessment with utility in this setting.

Cardiopulmonary exercise testing (CPET) has gained popularity as an objective measurement of “fitness” and is considered the gold standard for assessing physiological reserve. Lower anaerobic threshold and low peak VO_2_ have been associated with both pre‐LT mortality and with increased post‐LT intensive care length of stay (LOS) and mortality.^21^, ^22^, ^23^, ^24^, ^25^

Despite the availability of a wide number of physiological reserve tests to assist in prediction of post‐transplant outcomes, there is a lack of consensus about the most useful and cost‐effective test to use in routine care. For example, to the best of our knowledge, there are no studies comparing resource‐intensive gold standard tests (CPET) with simpler bedside test (handgrip strength, MAMC).

The aim of this study was therefore to compare these tests (CPET, handgrip strength, MAMC) performed during LT assessment in their ability to predict adverse outcomes after LT.

## Methods

This was a retrospective cohort study of patients who underwent liver transplant assessment and subsequent liver transplant at the South Australian Liver Transplant Unit, located at Flinders Medical Centre, Adelaide, South Australia. The South Australian Liver Transplant Unit provides LT services to a population of approximately 2 million people across South Australia and the Northern Territory. All patients who underwent LT between1 January 2015 to 31 August 2020 were eligible for inclusion in the study. All LT patients are followed up long‐term by the unit via both face‐to‐face and telehealth consults.

Patient information was collected from both paper and electronic medical record systems. Information on physiological reserve assessments, biochemistry, Model for End‐Stage Liver Disease (MELD), and Child–Pugh scores were recorded at the time of transplant assessment.

CPET was performed in a subset of 20 patients using a ramped exercise protocol aiming for 10 min of loaded exercise on a stationary bicycle with continuous monitoring of cardiac rhythm, expired gas, and pulse oximetry.^26^, ^27^ The following parameters were measured: lactate threshold, peak exercise oxygen uptake (peak VO_2_), and minute ventilation/carbon dioxide production (V_E_/VCO_2_) slope.

Handgrip strength was measured with a single spring dynamometer, as described by Klidjian *et al*.^28^ Three recordings were taken on each arm with at least 10 s rest between each, and the highest value recorded. Handgrip strength analyses were not divided into male and female cutoff values but were analyzed as a whole group. Subsequent multivariate analyses for both continuous and categorical (tertiles) handgrip strength data were adjusted for gender and other variables.

MAMC was calculated as described by Lohman *et al*.: MAMC = MAC − (3.14 × TSF thickness); where MAC is mid‐arm circumference and TSF is triceps skinfold.^29^

To reduce measurement variability, handgrip strength and MAMC were performed by the same operator (ward dietician), and CPET testing was performed by the same operator (respiratory physiologist) during the study period.

Length of ICU stay and overall LOS were calculated from the date of transplant to the date of discharge from ICU and hospital, respectively. Sepsis was defined as any severe infection requiring antibiotic treatment or hospital admission less than 12 months from liver transplant. Follow‐up time was taken from the date of transplant until death or 31 March 2021, whichever occurred earlier. There were no prehabilitation type interventions offered to patients on the waiting list beyond routine dietetic advice and management as appropriate for any patient with advanced liver disease.

### Statistical methods

Descriptive statistics were performed using frequency and percentages for categorical data and mean (SD) or median (interquartile range [IQR]) for normally and non‐normally distributed continuous variables. Univariate and multivariate binary logistic regression were used to assess 12‐month sepsis and death. Length of hospital stay and length of ICU stay were assessed using univariate and multivariate Cox regression models with the Breslow method for ties. A hazard ratio below 1.00 indicated longer LOS. The multivariate regression models were adjusted for age, sex (model 1) and fully adjusted for age, sex, MELD score, Child–Pugh score, etiology, and the Charlson Comorbidity Index (model 2). For the outcome “sepsis within 12 months” an additional analysis was performed using handgrip strength as a categorical variable (using tertiles) and using predictive margins for tertiles of handgrip strength. Adjusting for gender is necessary to ensure the physiological reserve–outcome associations are not simply a result of confounding due to there possibly being differences in physiological reserve according to gender. However, there may still be differences in the strength of the physiological reserve–outcome associations according to gender. An additional stratified analysis was performed to assess for this.

### Ethical approval

This study was approved by the Southern Adelaide Clinical Human Research Ethics Committee (AUD/20/SAC/211).

## Results

### Patients' characteristics

There were 126 transplants performed on 124 patients during the study period. There were 15 patients who lacked data points for any of the physiological reserve measurement variables, so they were excluded from the final analysis of 109 transplants in 109 individual patients. Patient characteristics are shown in Table 1. The median (IQR) age of transplantation candidates was 57 (50–63). Patients were predominantly male (66%), with a median (IQR) MELD score of 16 (10–20). The most common etiology of liver disease was combined hepatitis C virus and hepatocellular carcinoma (19.6%), followed by alcoholic liver disease (17.0%). The median (IQR) days between liver transplant assessment and LT was 165 (72–276).

**Table 1:** Patients' characteristics (*n* = 109)

Age, years (median, IQR)	57 (50–63)
MELD score (median, IQR)	16 (10–20)
Male gender, *n* (%)	74 (66%)
Liver disease etiology, *n* (%)	
Hepatitis C and HCC	22 (19.6)
Alcoholic liver disease	19 (17.0)
Other	12 (10.7)
NAFLD	10 (8.93)
Alcoholic liver disease and HCC	9 (8.04)
Autoimmune hepatitis	7 (6.25)
Primary sclerosing cholangitis	6 (5.36)
Primary biliary cirrhosis	5 (4.46)
NAFLD and HCC	4 (3.57)
Hepatitis C	4 (3.57)
Hepatitis B and HCC	4 (3.57)
Alcoholic liver disease and HCV	3 (2.68)
Hepatitis B	3 (2.68)
Acute liver failure	1 (0.893)
Time between liver transplant assessment and liver transplantation, days (median, IQR)	165 (72–276)
Handgrip strength, kg, (median, IQR) (*n* = 107)	33 (23.5–39)
Mid‐arm muscle circumference, cm, (median, IQR) (*n* = 109)	27 (24.5–30.6)
Lactate threshold, mL/min/kg, (median, IQR) (*n* = 20)	9 (7.35–9.575)
Peak VO_2_, mL/min/kg, (median, IQR) (*n* = 20)	14 (12.05–17.5)
VE/VCO_2_ slope, (median, IQR) (*n* = 20)	36 (31.75–40)

HCC, hepatocellular carcinoma; HCV, hepatitis C virus; IQR, interquartile range; MELD, Model for End‐Stage Liver Disease score; NAFLD, non‐alcoholic fatty liver disease; Peak VO_2_, peak exercise oxygen uptake; V_E_/VCO_2_ slope, minute ventilation/carbon dioxide production.

### Sepsis within 12 months

**Figure 1:**
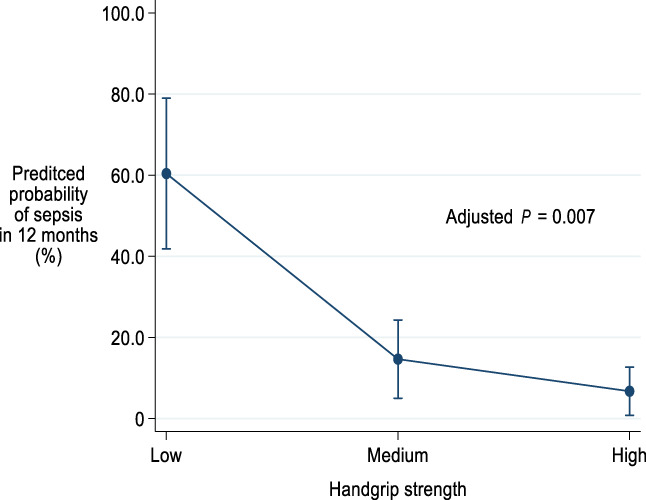
Marginal predicted^†^ probability (95% confidence interval [CI]) of severe sepsis at 12 months by tertile of handgrip strength. ^†^Adjusted for age, gender, Charlson Comorbidity Index, Model for End‐Stage Liver Disease score, Child Pugh score and aetiology of liver disease. Error bars represent 95% CI.

There were 22 episodes of severe sepsis in patients occurring within 12 months of LT. The results from univariate and multivariate logistic regression analyses for the association with sepsis within 12 months are shown in Table 2. There were no associations between sepsis and MAMC or CPET when assessed as continuous variables in univariate analysis or multivariate analysis. Handgrip strength was associated with sepsis within 12 months after adjusting for multiple confounders (odds ratio [OR] = 0.89; 95% confidence interval [CI] 0.82–0.98; *P* = 0.014). Tertiles of handgrip strength were a significant predictor of sepsis within 12 months (Table 3). Patients with handgrip strength in the lowest tertile were more likely to have sepsis compared with those in the middle (OR = 0.21; 95% CI 0.05–0.91; *P* = 0.036) or highest (OR = 0.14; 95% CI 0.03–0.74; *P* = 0.02) tertile when adjusting for age and gender. This relationship was also present when adjusting for multiple confounders for both the middle (OR = 0.012; 95% CI 0.001–0.292; *P* = 0.006) and highest (OR = 0.004; 95% CI 0.000–0.125; *P* = 0.002) tertiles. The observed rate of sepsis was higher in the lowest tertile (28.1%) compared with those in the middle (12.2%) or highest (10.8%) tertiles. The predicted probability of sepsis within 12 months, following full adjustment for patients in the lowest tertile for handgrip strength was 60.4%, for the middle tertile 14.6%, and the highest tertile 6.74% (Table 4, Fig. 1).

**Table 2:** Association of physiological reserve measures with sepsis within 12 months of liver transplantation

	Univariate analysis		Model 1^†^		Model 2^‡^	
	Odds ratio (95% CI)	*P* value	Odds ratio (95% CI)	*P* value	Odds ratio (95% CI)	*P* value
Handgrip strength, kg (*n* = 107)	0.96 (0.92–1.01)	0.130	0.94 (0.89–1.00)	0.065	0.89 (0.82–0.98)	0.014
MAMC (*n* = 109)	0.95 (0.84–1.08)	0.437	0.95 (0.84–1.09)	0.489	0.95 (0.81–1.12)	0.561
Lactate threshold, mL/min/kg, (*n* = 20)	1.04 (0.65–1.67)	0.857	0.61 (0.30–1.27)	0.185	Not estimable^§^	—
Peak VO_2_, mL/min/kg, (*n* = 20)	0.94 (0.74–1.19)	0.584	Not estimable^§^	—	Not estimable^§^	—
VE/VCO_2_ slope (*n* = 20)	1.15 (1.00, 1.32)	0.053	1.22 (1.00–1.49)	0.06	Not estimable^§^	—

^†^ Adjusted for age and gender.

^‡^ Adjusted for age, gender, Charlson Comorbidity Index, Model for End‐Stage Liver Disease score, Child–Pugh score, and etiology of liver disease.

^§^ Not estimable due to insufficient observations to permit modeling.

CI, confidence intervals; MAMC, mid‐arm muscle circumference; Peak VO_2_, peak exercise oxygen uptake; V_E_/VCO_2_ slope, minute ventilation/carbon dioxide production.

**Table 3:** Association with early (<12 months) sepsis after liver transplantation and handgrip tertiles (*n* = 107)

Tertile	*n*	Sepsis (%)	Univariate analysis		Model 1^†^		Model 2^‡^	
			Odds ratio (95% CI)	*P* value	Odds ratio (95% CI)	*P* value	Odds ratio (95% CI)	*P* value
1	31	9 (28.1)	1.00	—	1.00	—	1.00	
2	40	5 (12.2)	0.35 (0.10–1.19)	0.094	0.21 (0.05–0.91)	0.036	0.012 (0.001–0.292)	0.006
3	36	4 (10.8)	0.31 (0.09–1.13)	0.076	0.14 (0.03–0.74)	0.020	0.004 (0.000–0.125)	0.002

^†^ Adjusted for age and gender.

^‡^ Adjusted for age, gender, Charlson Comorbidity Index, Model for End‐Stage Liver Disease score, Child–Pugh score, and etiology of liver disease.

CI, confidence intervals.

**Table 4:** Marginal predicted probability^†^ of sepsis within 12 months of liver transplantation by tertile of handgrip strength

Tertile	*n*	Margin (95% CI)	*P* value
1	31	0.604 (0.418–0.790)	0.001
2	40	0.146 (0.0499–0.243)	0.003
3	36	0.067 (0.00789–0.127)	0.026

^†^ Using multivariate binomial logistic regression model with age, gender, Charlson Comorbidity score, Model for End‐Stage Liver Disease score, Child–Pugh, and etiology as included covariates.

CI, confidence intervals.

### One‐year mortality

There were six post‐LT deaths occurring within 12 months of the procedure, with 1‐year mortality rate post‐LT of 5%. Causes of death included disseminated cytomegalovirus/multi‐resistant pseudomonas infection, cryptococcal meningitis, cardiac arrest, heart failure, glioblastoma, and progressive thrombotic microangiopathy. Neither handgrip strength nor MAMC were associated with mortality within 12 months in univariate or multivariate analysis (Table 5).

**Table 5:** Association of physiological reserve measures with 12‐month mortality after liver transplantation

	Univariate analysis		Model 1^†^		Model 2^‡^	
	Hazard ratio (95% CI)	*P* value	Hazard ratio (95% CI)	*P* value	Hazard ratio (95% CI)	*P* value
Handgrip strength, kg (*n* = 107)	1.0 (0.93–1.07)	0.942	0.98 (0.90–1.06)	0.616	0.94 (0.80–1.11)	0.477
Mid‐arm muscle circumference, cm. (*n* = 109)	0.92 (0.76–1.10)	0.364	0.88 (0.71–1.08)	0.211	0.93 (0.65–1.33)	0.678

^†^ Adjusted for age and gender.

^‡^ Adjusted for age, gender, Charlson Comorbidity Index, Model for End‐Stage Liver Disease score, Child–Pugh score, and etiology of liver disease.

CI, confidence intervals.

### Hospital LOS post‐LT

The associations between hospital LOS and physiological reserve measures are shown in Table 6. Hospital LOS was associated with handgrip strength (hazard ratio [HR] 1.02; 95% CI 1.01–1.04; *P* = 0.007) on univariate analysis. This association persisted with full adjustment (HR = 1.03; 95% CI 1.00–1.06; *P* = 0.034). Lactate threshold was associated with hospital LOS (HR = 1.43; 95% CI 1.07–1.91; *P* = 0.017) after univariate analysis. When adjusting for age and gender, lactate threshold (HR = 1.55; 95% CI 1.10–2.17; *P* = 0.011) and peak VO_2_ (HR = 1.20; 95% CI 1.03–1.41; *P* = 0.023) were both associated with hospital LOS. Although a shorter hospital LOS post‐LT with increased lactate and peak VO_2_ was seen when adjusted for age and gender, this was not significant on multivariate analysis with full adjustment. Hospital LOS was not associated with MAMC on any analysis.

**Table 6:** Associations of physiological reserve measures with hospital length of stay

	Univariate analysis		Model 1^†^		Model 2^‡^	
	HR (95% CI)	*P* value	HR (95% CI)	*P* value	HR (95% CI)	*P* value
Handgrip strength, kg (*n* = 107)	1.02 (1.01–1.04)	0.007	1.02 (1.00–1.04)	0.063	1.03 (1.00–1.06)	0.034
Mid‐arm muscle circumference, cm (*n* = 109)	1.01 (0.97–1.06)	0.554	1.01 (0.96–1.06)	0.797	1.04 (0.98–1.11)	0.173
Lactate threshold, mL/min/kg (*n* = 20)	1.43 (1.07–1.91)	0.017	1.55 (1.10–2.17)	0.011	0.72 (0.39–1.32)	0.288
Peak VO_2_, mL/min/kg (*n* = 20)	1.09 (1.00–1.19)	0.056	1.20 (1.03–1.41)	0.023	1.07 (0.78–1.48)	0.662
VE/VCO_2_ slope (*n* = 20)	0.95 (0.88–1.01)	0.114	0.95 (0.88–1.01)	0.105	0.49 (0.24–1.00)	0.050

^†^ Adjusted for age and gender.

^‡^ Adjusted for age, gender, Charlson Comorbidity Index, Model for End‐Stage Liver Disease score, Child–Pugh score, and etiology of liver disease.

HR < 1.00 indicates longer length of stay.

CI, confidence intervals; HR, hazard ratio; Peak VO_2_, peak exercise oxygen uptake; V_E_/VCO_2_ slope, minute ventilation/carbon dioxide production.

### Intensive care LOS post‐LT

The associations with physiological reserve measures with intensive care LOS are shown in Table 7. Handgrip strength was significant on multivariate analysis when adjusted for age and gender but there was no statistically significant association on multivariate analysis with full adjustment. While all CPET measures were significant on adjustment for age and gender, on fully adjusted multivariate analysis the LOS in intensive care was shorter with increased peak VO_2_ (HR 1.83; 95% CI 1.06–3.16; *P* = 0.03) and longer with increased V_E_/VCO_2_ slope (HR 0.71; 95% CI 0.58–0.88; *P* = 0.002). ICU LOS was not associated with MAMC.

**Table 7:** Associations of physiological reserve measures with intensive care length of stay

	Univariate analysis		Model 1^†^		Model 2^‡^	
	HR (95% CI)	*P* value	HR (95% CI)	*P* value	HR (95% CI)	*P* value
Handgrip strength, kg (*n* = 107)	1.02 (1.00–1.03)	0.050	1.02 (1.00–1.04)	0.034	1.02 (1.00–1.05)	0.086
Mid‐arm muscle circumference, cm (*n* = 109)	1.02 (0.97–1.06)	0.467	1.02 (0.98–1.06)	0.441	1.04 (0.98–1.02)	0.215
Lactate threshold, mL/min/kg (*n* = 20)	1.25 (0.97–1.62)	0.079	1.40 (1.00–1.96)	0.049	1.36 (0.54–3.42)	0.515
Peak VO_2_, mL/min/kg (*n* = 20)	1.21 (1.07–1.38)	0.002	1.33 (1.12–1.58)	0.001	1.83 (1.06–3.16)	0.030
V_E_/VCO_2_ slope (*n* = 20)	0.91 (0.85–0.98)	0.013	0.91 (0.85–0.98)	0.009	0.71 (0.58–0.88)	0.002

^†^ Adjusted for age and gender.

^‡^ Adjusted for age, gender, Charlson Comorbidity Index, Model for End‐Stage Liver Disease score, Child–Pugh score, and etiology of liver disease.

HR < 1.00 indicates longer length of stay.

HR, hazard ratio; Peak VO_2_, peak exercise oxygen uptake; VE/VCO_2_ slope, minute ventilation/carbon dioxide production.

### Sensitivity analysis

In order to exclude any further association between gender and the outcomes, a further stratified analysis was performed, shown in Table 8. This was underpowered to detect any statistically significant associations.

**Table 8:** Stratified analysis^†^ (male/female gender) of association between handgrip strength and mid‐arm muscle circumference with outcome variables

	Odds ratio (95% CI)	*P* value
Sepsis (*n* = 34 for females and 73 for males)		
Females		
HGS	1.03 (0.85–1.25)	0.741
MAMC	1.00 (0.80–1.25)	0.99
Males		
HGS	0.93 (0.87–1.00)	0.055
MAMC	0.96 (0.80–1.15)	0.66
Hospital length of stay (*n* = 34 for females and 73 for males)		
Females		
HGS	1.03 (0.97–1.11)	0.307
MAMC	1.00 (0.91–1.10)	0.960
Males		
HGS	1.01 (0.99–1.04)	0.358
MAMC	1.03 (0.96–1.10)	0.410
Intensive care length of stay (*n* = 34 for females and 73 for males)		
Females		
HGS	1.08 (1.00–1.16)	0.053
MAMC	0.99 (0.91–1.08)	0.781
Males		
Male HGS	1.02 (1.00–1.05)	0.084
Male MAMC	1.05 (0.99–1.12)	0.124
Mortality (*n* = 34 for females and 73 for males)		
Females		
HGS	^‡^	^‡^
MAMC	^‡^	^‡^
Males		
HGS	0.91 (0.81–1.02)	0.093
MAMC	0.76 (0.57–1.01)	0.057

^†^ Adjusted for age, Charlson Comorbidity Index, Model for End‐Stage Liver Disease score, and Child–Pugh score.

^‡^ Not estimable due to only one female death.

CI, confidence intervals; HGS, Hand grip strength; MAMC, mid‐arm muscle circumference.

## Discussion

This study examined the predictive value of three physiological reserve measures that were routinely used at our center during the assessment of liver transplant candidates. The data demonstrated that both handgrip strength and CPET provided useful prediction for clinically important post‐LT outcomes. Specifically, CPET measures were independently associated with intensive care LOS, and handgrip strength was independently associated with hospital LOS and sepsis within 12 months. MAMC was not a useful instrument in this study and was not associated with any of the clinical outcomes assessed.

This study adds to the growing literature supporting the concept that physiological reserve measures have independent utility in predicting surgical outcomes,^30^, ^31^, ^32^ particularly in LT.^20^, ^21^, ^33^ The identification of poor physiological reserve in LT candidates has important implications. Firstly, it provides an opportunity for transplant units to intervene with preoperative prehabilitation interventions such as nutritional and exercise interventions. Such interventions may be useful in reducing adverse events post‐LT; however, evidence in this area is still developing.^34^, ^35^, ^36^ Secondly, the identification of poor physiological reserve may provide a warning to transplant teams about increased risk and futility of proceeding with marginal transplant candidates.

The data confirm the clinical usefulness of handgrip strength, a simple, single bedside test of reduced muscle strength. Handgrip strength was associated with multiple key outcome measures and this finding is consistent with the emerging concept that measures of functional muscle strength may be more clinically useful than those of muscle mass. Measures of muscle mass cannot provide information about quality of muscle, and it has been shown that the correlation between muscle strength and mass is only modest.^37^ Indeed, one of the few studies to compare handgrip strength with muscle mass was performed by Sinclair *et al*.,^33^ who demonstrated that handgrip strength, when combined with MELD score, was superior at predicting mortality over imaging‐based measures of sarcopenia in men with cirrhosis (20). The study findings are supported by other studies that have also demonstrated the utility of handgrip strength in other relevant settings including as a predictor of mortality in patients with chronic liver disease.^38^ A comparison between the performance of handgrip strength and psoas muscle mass for this cohort would have been informative, but data for psoas muscle mass were unfortunately not available. A comparison between the performance of handgrip strength and the Liver Frailty Index (hand grip strength, chair stands, and balance testing) would have also been informative, particularly as the Liver Frailty Index has been shown to be a more accurate predictor of muscle atrophy relative to handgrip strength.^39^ Unfortunately these data were not available for the cohort.

CPET measures were also useful in predicting intensive care LOS. These are measures of cardiorespiratory fitness and ventilatory efficiency, and the results demonstrate the significance of these factors for patients undergoing LT. CPET however has several limitations relevant to LT assessment. Firstly, it requires complex equipment and time resource, which may not be widely available for all transplant units. Secondly, a percentage of LT candidates are too unwell to perform this testing. In comparison, handgrip strength is simple, inexpensive, and can be performed at the bedside without the need for complex training or significant time allocation during a busy transplant workup schedule. It was also found to be associated with multiple significant post‐LT early adverse outcomes (sepsis, hospital LOS, ICU LOS), whereas CPET was associated with only one (ICU LOS). Due to these factors, handgrip strength would appear to provide greater clinical utility and cost‐effectiveness as a tool for the assessment of physiological reserve in LT candidates. To the best of our knowledge, this is the first study to compare the clinical utility of both handgrip strength and CPET measures and their ability to predict post‐LT adverse outcomes.

The underlying biological mechanisms by which frailty and sarcopenia contribute to poor clinical outcomes in this setting are unknown. However, several plausible mechanisms have been proposed, including the involvement of “myokines,” which are cytokines or peptides produced during skeletal muscle contraction. Irisin, a myokine that is released during exercise and improves mitochondrial function, is suppressed in sepsis.^40^ Myostatin is another myokine and is a negative regulator of muscle protein synthesis.^41^ These have been implicated in liver disease.^40^, ^41^ One myokine, fractalkine/CX3CL1, has been shown to be significantly upregulated by a low‐intensity resistance exercise program.^42^ Measurement of these myokines at baseline and correlation with clinical outcomes after LT would be an interesting further study to investigate the utility of myokines as alternatives to current measures.

Our study had several limitations. Firstly, CPET measures were only available in 20 patients, as CPET was introduced for LT candidates from 2018. This limited the statistical power of the results for these variables. In addition, there was a low number of deaths during the first post‐transplant year, and consequently limited statistical power to detect associations of our physiological reserve measures with 1‐year mortality. Despite these limitations, we were able to find several statistically significant associations for the majority of outcome variables.

Interpretation of study findings is also limited by the single‐center, retrospective observational design of the study. For example, the accuracy of data collection for sepsis events was limited to events recorded within the medical record databases. We therefore cannot exclude the possibility of missed cases of sepsis that occurred outside our hospital networks that may have led to underreporting of this outcome. As with all non‐randomized studies, there was potential for important unmeasured residual confounding. However, this risk of bias was reduced by the statistical analyses adjusting for known potential confounders.

Despite these limitations, we believe the study provides useful information for LT teams and is likely to be generalizable to typical LT candidates given the limited exclusions from the study (largely patients with acute liver failure without physiological reserve assessments). This pilot study provides justification for larger, prospective, multicentred studies comparing the relative utility and cost‐effectiveness of CPET and handgrip strength during LT assessment.

In conclusion, Handgrip strength and CPET in liver transplant candidates both provided clinically useful prediction of important adverse events following LT. Low handgrip strength was associated with early post‐LT sepsis and prolonged hospital stay on fully adjusted analysis. Poor performance at CPET was associated with prolonged intensive care stay on fully adjusted analysis. Particularly in low resource settings, handgrip strength is a useful tool to identify LT candidates with higher risk of early adverse events following transplantation.
